# The evaluation of phenylalanine levels in Estonian phenylketonuria patients during eight years by electronic laboratory records

**DOI:** 10.1016/j.ymgmr.2019.100467

**Published:** 2019-03-23

**Authors:** Hardo Lilleväli, Karit Reinson, Kai Muru, Siret Saarsalu, Kadi Künnapas, Tiina Kahre, Ülle Murumets, Katrin Õunap

**Affiliations:** a Institute of Biomedicine and Translational Medicine, University of Tartu, Tartu, Estonia; b Department of Clinical Genetics, United Laboratories, Tartu University Hospital, Tartu, Estonia; c Department of Clinical Genetics, Institute of Clinical Medicine, University of Tartu, Tartu, Estonia

**Keywords:** Phenylketonuria, Blood phenylalanine value, Adherence to treatment, Electronic laboratory records, PKU, phenylketonuria, Phe, phenylalanine, PAH, phenylalanine hydroxylase, HPA, hyperphenylalaninaemia, UL-TUH, United Laboratories of Tartu University Hospital, BH_4_, tetrahydrobiopterin, LIMS, laboratory information management system, NGO, non-governmental organisation

## Abstract

Blood phenylalanine (Phe) values from the dried blood spots of all Estonian phenylketonuria (PKU) patients have been deposited into a unified electronic laboratory database for eight years, providing an opportunity to assess the adherence of the patients to dietary recommendations over time and to observe patient practices both individually and collectively. Our results demonstrate generally good adherence to clinical and dietary recommendations during the first six years of life, as the percentage of patients with median Phe values fitting under the national recommendation levels were 95%, 84% and 70% in age groups 0–1, 1–2 and 2–6 years, respectively. Conversely, significant deviations occur in the group of 6 to 12 year-olds, mildly decreasing in adolescence and increasing in adulthood (43%, 53% and 57%, respectively). Wide individual differences occurred in all groups, especially in patients with a classical PKU phenotype caused by *PAH* variants that fully abolish phenylalanine hydroxylase activity. Surprisingly, some of the best dietary adherence was seen in the late-diagnosed PKU patients with poor cognitive functioning. As a rule, the median of Phe values crosses the recommended thresholds in approximately one third to one half of the patients of each age group after the first two years of life.

## 1 Introduction

Phenylketonuria (PKU, OMIM #261600) is a classic example of an inborn error of metabolism, treatable with careful dietary management with a good clinical outcome. Classical PKU is caused by the deficiency of phenylalanine hydroxylase (PAH, EC 1.14.16.1) resulting in accumulation of excess phenylalanine (Phe) from dietary protein and causing the condition of hyperphenylalaninaemia (HPA), which is the main cause of neurological disturbances and intellectual disability in cases of untreated PKU [1]. While in unaffected individuals the Phe levels do not exceed 120 μmol/L (usually lower) [2], the safe and recommended values for those with PKU have been generally accepted to be three- to fivefold higher, depending on age.

Despite the fact that this treatment has been available for decades, adherence to recommended dietary restrictions has been always an issue of concern, as everyday maintenance of the diet together with social and economic issues may cause deviations from the ideal practice.

In 2017, European guidelines for the diagnosis and management of patients with PKU were agreed upon and published after substantial work of a group of experts [3,4]. National guidelines of treatment, diagnostics and management of PKU had already been approved in Estonia in 2012 [5], providing approximately similar suggestions (See Table 1 for comparison [[3], [4], [5], [6], [7], [8]]). It is currently unclear how well these recommendations have been implemented by the PKU patients and their families in Estonia.

**Table 1:** Recommendations for follow-up of Phe values in PKU patients according to Estonian [5], European [4], US [6,8] and Australian [7] guidelines.

Age group	Recommended highest Phe value according to Estonian (EST) guidelines (μmol/L)	Recommended frequency for dietary follow-up - EST	Recommended highest Phe value according to European (EU) guidelines (μmol/L)	Recommended frequency for dietary follow-up - EU	Recommended highest Phe value according to United States (US) guidelines (μmol/L)	Recommended frequency for dietary follow-up - US	Recommended highest Phe value according to Australian guidelines (μmol/L)
0–2 y	0–12 months – 4; 240	0–12 months- weekly	360	0–12 months- weekly	360	0–12 months- weekly	360
	1–2 y − 240 (max 360)	1–2 y – twice per month		1–2 y – twice per month		1–2 y – twice per month	
2–12 y	240 (max 360)	2–6 y – monthly	360	Twice per month	360	Once to twice per month	360
		7 -12y – 4 to 6 times yearly					
12–18 y	360 (max 600)	4 to 6 times per year	600	Monthly	360	Monthly	500
Adult (18+ y)	360 (max 600)		600	Monthly	360	Monthly	500

Since 2010, all results of any biochemical analysis performed in the United Laboratories of Tartu University Hospital (UL-TUH) have been recorded in a unified electronic laboratory information management system (LIMS), enabling an overview of all analyses preformed in every patient (incl. Phe measurements). Herewith we have created and analysed a sub-database from LIMS to obtain a generalized overview of the maintenance of dietary therapy and established practice of medical observation of Estonian PKU patients.

## 2 Material and methods

### 2.1 The database of PKU and HPA patients

We created a sub-database of all available entries for the period 2010 to March 2018 from the general LIMS database available for any diagnostic analysis performed in UL-TUH, concerning all Estonian patients diagnosed with PKU or other forms of HPA. This database included the following fields: name, personal ID-code, date of sample collection, age at the moment of sample collection, assignment to age group at the moment of sample collection, Phe value in dried blood spot (either mg/dL or μmol/L), genotype, phenotype, diagnosis time (either from the newborn screening or late diagnosis in case of persons born before 1993), and assessment of educational level (Table 2).

**Table 2:** Genotype, phenotype and Phe value data of Estonian PKU patients included in the study.

Patient code	Genotype	Phenotype	Diagnosed	Education level (ISCED 2011) or current education	Pre-treatment max Phe μmol/L	Total entries	min Phe μmol/L	max Phe μmol/L	Phe median abs μmol/L
BH	p.R408W/p.R408W	Classical	Screening	4	1585	14	366	817	648
DJ	p.R408W/p.R408W	Classical	Screening	Preschool age	1543	114	20	570	181
DI	p.R408W/p.R408W	Classical	Screening	Preschool age	1380	212	61	866	193
DC	p.R408W/c.1315 + 1G > A	Classical	Screening	Assisted education	2623	35	54	1399	551
BG	p.R408W/p.R408W	Classical	Screening	6	1543	26	333	831	562
CO	p.R408W/c.1315 + 1G > A	Classical	Screening	Normal school	1446	287	54	957	182
CK	p.R408W/p.R408W	Classical	Screening	Normal school	1137	49	387	1414	775
CP	p.R408W/c.1315 + 1G > A	Classical	Screening	Normal school	2077	110	54	690	107
DO	p.R408W/p.R408W	Classical	Screening	Preschool age	1313	89	6	694	177
BC	p.R408W/p.R408W	Classical	Late diagnosed	2	NA	14	222	1235	602
DB	p.R408W/p.R408W	Classical	Screening	Normal school	1616	103	54	1520	551
DL	p.R408W/p.R261Q	BH4-sensitive	Screening	Normal school	1471	29	23	674	266
DG	p.R408W/p.R261Q	Classical	Screening	Preschool age	1532	122	34	593	164
BN	p.R408W/p.D222*	Classical	Screening	3	1405	12	593	1066	969
AF	p.R408W/p.R408W	Classical	Late diagnosed	1	890	14	10	109	54
CB	p.R408W/p.A300S	Benign HPA	Screening	4	NA	5	115	254	133
CA	p.R408W/ND	Mild	Screening	4	291	22	200	678	345
BL	p.R408W/p.I306V	Mild	Screening	6	400	40	133	432	227
AN	p.R408W/p.R408W	Classical	Late diagnosed	2	NA	41	382	1162	781
BE	p.R408W/p.R408W	Classical	Late diagnosed	1	1452	12	54	751	119
BB	p.R408W/p.R408W	Classical	Late diagnosed	3	NA	105	19	981	514
BJ	p.R408W/p.R252W	Classical	Late diagnosed	1	2305	18	436	1368	787
CD	p.R408W/p.R408W	Classical	Screening	4	1822	199	25	923	327
AA	p.R408W/p.R408W	Classical	Late diagnosed	3	NA	94	54	1197	454
BI	p.R408W/p.R408W	Classical	Screening	4	1017	176	54	1118	506
AM	p.R408W/p.R408W	Classical	Late diagnosed	1	NA	34	54	636	173
AB	p.R408W/p.R408W	Classical	Late diagnosed	3	NA	41	54	726	375
BM	p.R408W/p.R408W	Classical	Screening	5	1762	24	630	1368	884
CL	p.R408W/p.R408W	Classical	Screening	Normal school	1411	14	54	799	179
DP	p.L48S/p.E280K	Classical	Screening	Preschool age	314	23	39	179	92
CG	p.R408W/p.A300S	Benign HPA	Screening	Normal school	NA	5	157	200	163
DD	p.R408W/p.L48S	BH4-sensitive	Screening	Normal school	720	196	54	896	248
BF	p.R408W/p.S349P	Classical	Late diagnosed	6	2160	109	54	896	424
CF	p.R408W/p.E280K	Classical	Screening	Normal school	2185	98	61	1302	569
DA	p.L48S/p.E280K	Classical	Screening	Normal school	1544	119	54	769	182
CN	p.R408W/p.R408W	Classical	Screening	Normal school	2216	81	73	811	448
DN	p.R408W/p.R408W	Classical	Screening	Preschool age	571	93	8	786	101
BD	p.R408W/c.1315 + 1G > A	Classical	Late diagnosed	2	NA	2	1150	1168	1159
CE	p.R408W/p.R408W	Classical	Screening	Normal school	2149	23	258	1023	551
AD	p.R408W/p.R408W	Classical	Late diagnosed	1	NA	9	206	757	437
DE	p.R408W/p.R408W	Classical	Screening	Preschool age	2403	311	8	914	85
CJ	p.R408W/p.R408W	Classical	Screening	Normal school	1846	79	54	957	357
DM	p.R408W/p.R408W	Classical	Screening	Preschool age	609	77	7	564	93
CM	p.R408W/p.R261Q	BH4-sensitive	Screening	Assisted education	1501	118	54	790	347
AH	p.R158Q/c.1315 + 1G > A	Classical	Late diagnosed	2	NA	30	345	914	533
DQ	p.R408W/p.R408W	Classical	Screening	Preschool age	1213	24	12	590	36
CI	p.R408W/p.E280K	Classical	Screening	Normal school	2355	94	54	880	412
BK	p.R408W/p.L48S	Classical	Screening	4	1211	36	297	714	490
BA	p.R408W/p.R408W	Classical	Late diagnosed	3	NA	86	54	490	150
AL	p.R408W/p.R408W	Classical	Late diagnosed	1	NA	1	890	890	890
DF	p.R408W/p.I65T	Classical	Screening	Preschool age	2282	75	52	513	139
AJ	p.R408W/p.R408W	Classical	Late diagnosed	3	NA	24	54	1108	932
DK	p.R408W/p.P281L	Classical	Screening	Preschool age	2785	158	54	1174	559
AK	p.R408W/p.R408W	Classical	Late diagnosed	1	1845	6	176	611	490
CH	p.R408W/p.R408W	Classical	Screening	Normal school	2730	14	285	733	552
AI	p.R408W/p.R408W	Classical	Late diagnosed	2	NA	70	13	969	97
DH	p.R408W/p.E390G	BH4-sensitive	Screening	Preschool age	351	51	54	696	224
AG	p.R408W/p.R408W	Classical	Late diagnosed	1	NA	12	304	1168	562
AE	p.R408W/p.R408W	Classical	Late diagnosed	3	1616	149	54	1616	642
AC	p.R408W/p.R408W	Classical	Late diagnosed	2	NA	9	579	1392	756
				Medians:		41	54	848	394

NA – not available.

The initial created database included 4290 entries from 69 patients. All patients were carefully classified by genotype/phenotype data and these results have been previously published [9]. All data from individuals with initially suspicious samples obtained by newborn screening, but not confirmed with HPA, were excluded, as well as Phe levels confirming the newborn screening results and data obtained from a Phe loading/cofactor tetrahydrobiopterin (BH_4_) test. The information about BH_4_ responsiveness testing has been published earlier [9]. As the objective of the study was to draw insight into the quality and trajectory of ongoing therapy, nine of the subjects and all of their blood Phe values were excluded from the database due to late diagnosis associated with deep intellectual disability and/or refusal of treatment. The final database for further analysis therefore consisted of 4236 entries from 60 patients.

The following age groups were created: ≤1 year; 1 year 1 day to 2 years; 2 years 1 day to 6 years; 6 years 1 day to 12 years; 12 years 1 day to 18 years; >18 years (for simplicity further referred to as: 0–1 y, 1–2 y, 2–6 y, 6–12 y, 12–18 y, 18+ y, respectively). The age group 0–1 y contained 662 entries from 19 patients, 1–2y contained 548 entries from 19 patients, 2–6 y contained 1140 entries from 20 patients, 6–12y contained 470 entries from 19 patients, 12–18 y contained 477 entries from 16 patients and the adult group 18+ y contained 933 entries from 27 patients. We had only one patient (AE) with maternity in the adult group 18+ y; she had two pregnancies with strict diet during this study period. Median, maximum and minimum Phe values were counted for each group. All entries with Phe values above maximum recommended value of 360 μmol/L in case of patients up to 12 years of age and higher than 600 μmol/L in case of patients older than 12 years of age were counted and the ratio of entries elevating the recommended level was calculated.

### 2.2 Phenylalanine measurement

During the period under observation, two different methods of Phe measurement from dried blood spots (BS) collected on filter paper (Schleicher and Schuell filter paper No 2992) were used. These included the modified McCaman and Robins quantitative fluorescence-based method measuring ninhydrin-phenylalanine complex enhanced by L-leucyl-L-alanine dipeptide [10] measured on FluoroScan™ (Labsystems Oy, Helsinki, Finland) device using Labsystems neonatal phenylalanine kit (no. 6199 897) and LC-MS/MS tandem mass spectrometry on Waters Aquity™ Ultra Performance LC device using ChromSystems MassChrom® Amino Acids and Acylcarnitines from Dried Blood kit (order nr 55,000) applying neutral loss scan 120 detection, according to the methods provided by the manufacturer. Both methods were regularly evaluated for inner quality as well as by external quality controls and before the launch of the extended newborn screening in 2015 with LG-MS/MS, both methods were used in parallel for a couple of months and exhibited good correlation. Due to the switch in analytical methods in 2015 from fluorescence measurement with FluoroScan to LC-MS/MS analysis, Phe values were presented in different units. However, in order to unify the results, the values presented in mg/dL were converted (by multiplying by a factor of 60.54) to obtain unified numeric values. As the values obtained by FluoroScan were not distinguished if lower than 1 mg/dL and higher than 25 mg/dL (shown as <1 and >25 mg/dL in the original database, respectively), said values were substituted with 0.9 and 25.1 mg/dL, and further converted to μmol/L to enable analysis with numeric values. In order to avoid distortion in data analysis, we used median values in calculations instead of mean values, as the lower (<1 mg/dL) and higher (>25 mg/dL) result values obtained by McCaman-Robins method would have introduced too big error into finding the mean values, but were correctly interpreted in counting median values.

### 2.3 Assessment of educational level

Data about the educational level of the PKU patients were obtained during regular visits to outpatient clinic from the patients and/or their families. The patients younger than 18 y of age were assigned as “normal school”, “assisted education” or “preschool age”. In the patients older than 18 y of age, educational levels were evaluated according to ISCED 2011 scaling [11].

### 2.4 Data analysis

Data from LIMS were selected and analysed using Microsoft Excel software.

### 2.5 Compliance with ethical standards

This study was approved by Research Ethics Committee of the University of Tartu (approval date 21.09.2015 number 251/T-6).

## 3 Results

**Fig. 1:**
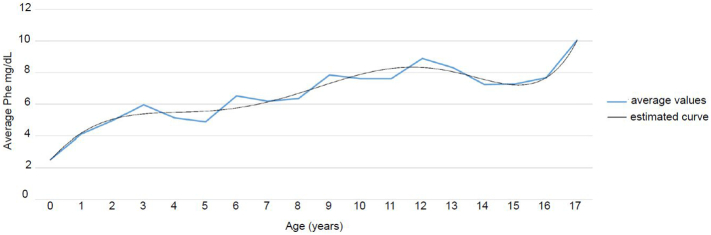
Average Phe values of all Estonian PKU patients of ages 0 to 18 years together during 2010 to 2018.

Among all of Estonian PKU patients, the median of medians of Phe values during the observed period was 394 μmol/L, reflecting that approximately half of the patients were able to sustain the recommended dietary treatment for half of the measurement instances (Table 2). The summary of the average Phe levels for the whole cohort is given in Fig. 1 (individual averages are presented in Supplementary Fig. 1). Only four of the 60 patients (patients AF, BA, CG, DP,) never exceeded the recommended Phe level during the entire evaluation period. Of these, only patient CG has the benign PKU phenotype, with the other three having classical PKU. As this assessment took into account all entries without discrimination of the age of the patient, the regularity of observations, or concomitant health problems, we therefore next split the database into more distinct six age groups.

**Fig. 2:**
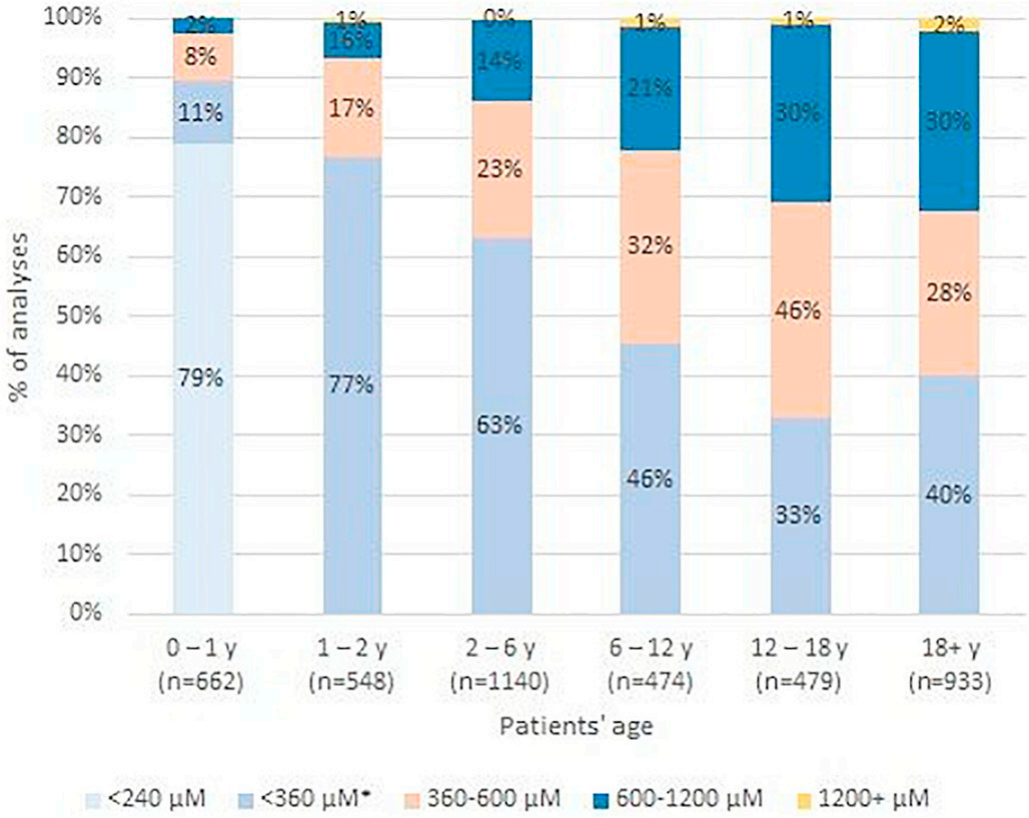
All entries (*N* = 4236) of blood spot Phe values of Estonian PKU/HPA patients in LIMS of UL-TUH are shown on a diagram regarding recommended limits as percentage along age groups. The diagram presents generalized overview of adherence to dietary recommendations in particular age groups and draws out the proportion of samples in well-managed patients and cases with lower dietary adherence.

**Fig. 3:**
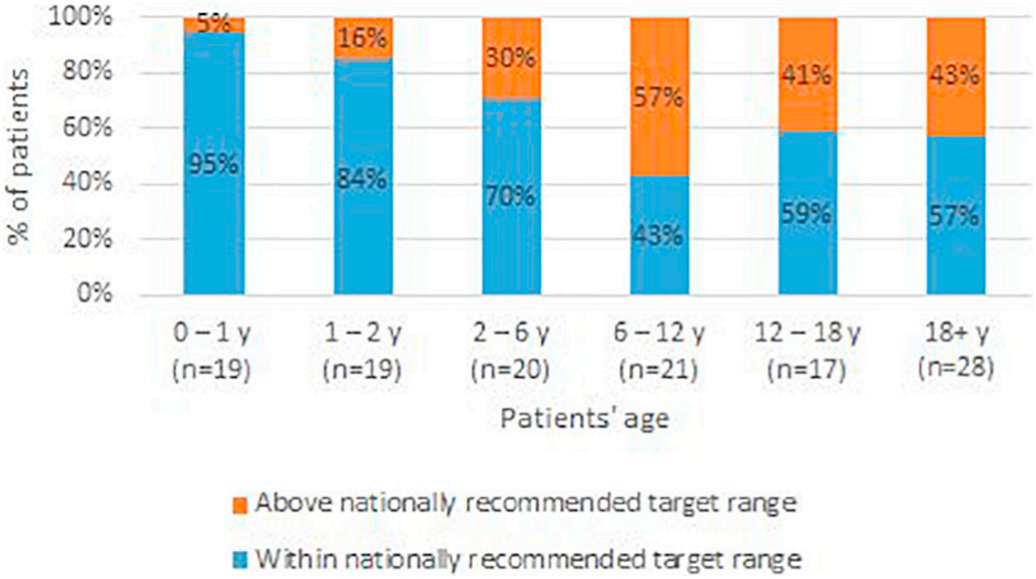
Median blood spot Phe values of Estonian PKU/HPA patients (*N* = 60) are shown in the diagram as percentage fitting under the recommended national values according to age groups. Here the medians of gathered blood spot values of each patient are assembled into the graph to present the proportion of well-managed individual diet among every age group.

All values of blood spot Phe analyses presented in relation to the recommended cut-off values in Estonia (see Table 1) are shown in a diagram (Fig. 2). All data are presented in groups by age of the patients. Fig. 3 shows the percentage of patients in an age group with median Phe values falling below the national recommendation levels of the respective age.

Results of the age group of 0–1 y (19 patients, Estonian reference value 240 μmol/L; European reference value 360 μmol/L – (results given in brackets); Table 1 Suppl). For 79% of all measurements and 95% of the patients, the median Phe values were within the range of the nationally-recommended levels (Fig. 2, Fig. 3). There were three (five) patients (CO, DP, DE), all with classical PKU, whose Phe value never exceeded the recommended level, while for six (nine) patients the elevated levels occurred in <10% cases of measurement, likely referring to occasional fluctuations possibly due to infections or random uncontrolled ingestions of unchecked products. In three patients, the elevated levels occurred between 10 and 20% of measurements, while four patients exhibited elevated levels in 50, 44 and 35% of measurements, which may indicate that the latter families were not able to consistently follow the advised dietary instructions. The median number of samples arriving to the laboratory was 35, which is lower than the recommended weekly frequency.

Results of the age group of 1–2 y (19 patients, Table 2 Suppl). These results exhibited some change compared to 0–1 y: for 77% of all measurements and 84% of the patients, the median Phe values were in the range of the nationally-recommended levels (Fig. 2, Fig. 3). The median of medians of Phe values remained at 224 μmol/L, suggestive of generally acceptable adherence to diet. The group contained the same 19 patients as the 0–1 y cohort, however in only three patients (CP, DE, DN) did the Phe value exceed suggested recommendations (360 μmol/L) in no >10% of measurements, while six patients exhibited elevated levels in >25% of the measurements, including two patients whose Phe values did not fit into the recommended level for a single case during this age period. Similarly to the first age group, the median count of samples per patient was 32, being in good accordance with the fortnightly recommendation.

Results of the age group of 2-6y (20 patients, Table 3 Suppl).For 63% of all measurements and 70% of the patients, the median Phe values were in the range of the nationally-recommended levels (Fig. 2, Fig. 3). The median of medians of Phe values was 285 μmol/L, but on the background of drastically diverging individual scores. In only four (patients DA, DE, DF, DH) did elevations above the recommendation (360 μmol/L) occur in <10% of cases; three of them having classical PKU and one has BH_4_-sensitive PKU. In three patients, the elevated levels occurred between 10 and 25% of measurements (patient CO, CP, DG), and all the remaining 13 patients exceeded the level more frequently, with four of them exceeding the level in 90–100% of entries, reflecting severe difficulties in keeping the diet. Additionally, the sampling frequency deviates substantially.

Results of the age group of 6–12 y (19 patients, Table 4 Suppl). For 46% of all measurements and 43% of the patients, median Phe values were in the range of the recommended levels (Fig. 2, Fig. 3). The tendency for elevated Phe increased as shown earlier (recommended 360 μmol/L). For only three cases of 19 patients was an elevated Phe level observed in <10% of entries, including one patient with mild HPA. Simultaneously, 11 of the patients had Phe increase above 360 μmol/L in >50% of analysed samples. This high ratio of elevated results may refer to difficulties in maintaining the dietary regimen the in rapidly changing social context of early school years. Blood spot sampling frequency also declined in this age group, as the median number of samples per patient was only 14 during six years.

Results of the adolescent group of 12–18 y (16 patients, Table 5 Suppl). For 79% of all measurements and 59% of the patients, the median Phe values were in the range of the nationally-recommended levels (Fig. 3). The distribution of elevated values among patients was notably more variable than in the younger groups: while in half of the patients, the ratio of elevated Phe samples remained in the limits from 0 to 40% of measured samples; another half exceeded the recommended level in more than half of the measured cases. Here we also observed relatively low activity in sending blood samples, as the median count was 14.5.

In the adult group of 18+y (27 patients, Table 6 Suppl). For 68% of all measurements and 57% of the patients, the median Phe values were in the range of the nationally-recommended levels (Fig. 3). For three patients (AF, BA, BL) the Phe value never exceeded the recommended level, two of them having late-diagnosed classical PKU phenotype, and one with a mild HPA; in five cases it happened only once or twice (AI, AK, AM, BE; all with classical phenotype, late diagnosis and p.Arg408Trp / p.Arg408Trp genotype; and BK from screening, classical phenotype), not taking into account those who presented their samples very rarely. Still, in 11 patients the elevated values were present in more than half of the measurements. Wide differences occurred in the frequency of sending control samples: from a single sample during the whole period to constant monitoring with 100–150 samples presented, resulting in a frequency of sampling of up to 14 to 18 times per year.

## 4 Discussion

Maintaining acceptable blood Phe levels in PKU patients as suggested in national guidelines [5,6] or more widely agreed among international consortiums [3,8] can be burdensome for families [12], even though it is clear that adherence to these recommendations is important for avoiding undesirable neuropsychiatric symptoms as well as intellectual disability [13,14]. Our results from the Estonian cohort of PKU patients reflect the tendencies of gradually occurring deviation from the suggested recommendations over time (Fig. 1). Our data present results from each patient during the eight year period (Supplementary Fig. 1), enabling analysis of the characteristics of dietary management both at the individual and group level.

Previously, similar observations have been presented by Walter et al., 2002 [12], wherein about a quarter of all samples from ages 0–4 years and 5–9 years exceeded the recommended level. Similarly to Walter et al. [12], our results show that blood Phe concentration was not always maintained below the recommend value.

The adherence to the recommendations in younger age groups remains remarkably higher, while with the increase of age and in adolescence the results became increasingly divergent. A report involving data from ten European PKU centres has shown similar results, reflecting that these same obstacles are to be faced in any country and population [15]. Ahring et al., 2011, has demonstrated that blood Phe concentrations increase with age, and we observed a similar tendency (Fig. 1), although we also observed a small decrease in average Phe concentrations at the age of 14 to 16 years that is not fully understood. One possible explanation is that dietary control in adolescents may be better than reported previously [15]. In comparison with the work of Jurecki et al., 2017 [16], our data also show better compliance with the recommendations in our adolescent patients than the pre-adolescence age group.

Our approach in the current study was to observe each of the patients during the entire available period, up to eight years. The median Phe value for either the whole period or selected age gives a better understanding of a particular patient's general adherence to dietary management if fluctuations in Phe over the recommended level remain sporadic. However, if the median value is observed to be higher than the recommendation, the family may warrant closer attention and observation.

During the first year of life, only a few families were not able to follow the dietary requirements, while most of the patients had only single occurrences of elevated Phe levels that were explainable by occasional infections or random dietary indiscretions. In general, our results depict the willingness of the families to follow the requirements as well as good level of clinical advice and dietary consultation.

The age group from 1 to 2 years already exhibited already more deviation from desirable Phe levels, though the general adherence rate still remained acceptable, as the median of median Phe values among the whole group remained 224 μmol/L, referring to the fact that more than half of the patients could maintain the desired levels in more than half of cases. This can still be considered reflective of good of parental control and family education. The results from the group of 2 to 6 year old children diverged, and the number of patients with only a few exceeded Phe values decreased, though the median of medians of Phe values remained lower than the recommended level. The next age group (6 to 12 y) faces the change of lifestyle and social activity: social pressure from school and more challenges in everyday life. Simultaneously, it may reflect the inability of school catering to comply with the needs of the children with special requirements. In the group of adolescents (12–18 y), differences in the regularity of follow-up, i.e. sample collection, become especially obvious. In spite of the relaxed recommendation of 600 μmol/L Phe level, nearly half of the patients exhibited elevated Phe levels in more than half of the cases. The same phenomenon is true about the adult group. Here we have not isolated the cases where female patients have become pregnant and therefore had stricter regulations of the diet. One reason for the difficulty of maintaining the Phe levels in blood in the observed patients may be the severity of particular *PAH* mutation, as the p.Arg408Trp variation vastly predominant in Estonian population [9] completely abolishes PAH activity, if present in homozygous state.

We have also focused on the data from patients with exceedingly elevated Phe levels: nine patients had a median Phe level of 720 μmol/L or higher. Phenotypically, they all exhibit the classical PKU phenotype, which is in accordance with their genotype, harbouring the predominant p.Arg408Trp variation of the *PAH* gene in one or both of the alleles, and in compound heterozygotes the second allele (p.Asp222*, p.Arg252Trp, c.1315+1G>A) has been shown to have a deleterious effect on PAH activity. Six of these patients had been diagnosed late, before the launch of national screening program, providing an explanation to their inability to adhere to the recommendations, as elevated Phe during their infancy had already caused cognitive damage. However, three remaining patients were not diagnosed late and exhibited normal progress in education (remarkably, patient BM has even succeeded achieving level 5 educational stage according to ISCED 2011 standards). This last example confirms the importance of early diagnosis and initiation of treatment, where even a relaxed attitude towards the diet in adolescence has not contributed negatively to educational achievement.

As would be presumed, the eight patients with mild or benign HPA, as well as with BH_4_-sensitive PKU, exhibit good adherence to the diet, with median Phe values clearly below recommended reference value, though still with occasional elevated fluctuations in case of patients DD and CM.

The possibility, that there has been born a person with mild HPA without being registered and medically supervised is improbable since the introduction of the screening programme in 1993 [17]. However, we cannot exclude the option that there may exist some adult patients born before 1993 with mild HPA who have never reached the scope of medical doctors. While Estonian PKU cohort has been shown to be genetically very homogenous [9], this chance is fortunately very low.

Moreover, during the last decades we have successfully implemented regular nutrition-practicing camps and courses organised by the Estonian PKU Association, an NGO bringing together families including a member with PKU. We have also been able to include regular consultations from a dietician since early infancy, and we presume this assistance may be a key factor for the families to adhere successfully to the recommendations.

The variability of dietary adherence was not connected to the severity of the genotype, as 20 of the patients with median Phe values under the recommended reference have the *PAH* genotype fully depleting PAH activity (14 of them are homozygotes for the p.Arg408Trp variant). However, the same severe mutations occurred in the patients with poor adherence. Surprisingly, five of the late diagnosed patients with low educational results manifest really well-controlled Phe levels, probably referring to well established family support or institutional care.

There are only four patients (AF, BA, CG, DP) in our cohort whose Phe values have been constantly under the reference value: one with benign HPA, other with the classical PKU version, while two of the latter belong to the group of late-diagnosed patients.

As another observation, the PKU patients of the same family (patients CI and CF; DA and DP; BC and AG) exhibit usually very similar Phe level patterns; in one pair (CL and CH) the divergence was greater and, interestingly, in one pair the late-diagnosed sibling (BA) has excellent dietary adherence, while his sister (BI) with a more timely diagnosis demonstrated more fluctuations and higher median Phe value.

There are only two centres for treating PKU in Estonia and some patients must travel a long distance for regular clinic visits. However, nowadays patients and/or their parents are may obtain Phe samples locally and send them to the laboratory by courier or by mail, receiving results mostly by email and occasionally by telephone call. We did not analyse our data by geographical distribution of the patients, though Freehauf et al., 2013 [18], has shown that geographic access to care does not impact control of Phe levels, but it does affect the number of monitoring samples sent to the clinic. We did observe that the patients with good adherence to the diet also had a tendency to perform regular sampling, and this should not be dependent on their place of residence.

## 5 Conclusions

We have provided insight into the dietary control measurements of an Estonian PKU patient cohort over an eight year period. These data have enabled our observation of both individual adherence to dietary management as well as displaying general tendencies characteristic to each age group.

Overall, we observed that during two first years of life, the families show good dietary adherence and follow the recommendations, with the exception of only a few families. However, the number of the cases of elevated Phe values subsequently increases with age, especially during early school age (6 to 12 y). In adolescence the picture slightly improves, but the relaxed dietary threshold for adults is still frequently crossed by most patients.

The ability to maintain the diet among the patients with classical PKU phenotype shows great variability, though, surprisingly, good results were seen among a few late-diagnosed patients, even those with poor cognitive functioning.

Our data reflect similar tendencies observed previously in other studies from different PKU management centres, an increase in cases of elevated Phe levels is seen in parallel with age.

The following are the supplementary data related to this article.

Supplementary Fig. 1 Individual yearly average phenylalanine (Phe) values of Estonian phenylketonuria patients. Supplementary Fig. 1

Supplementary Table 1 Maximal, minimal, and median values of Estonian PKU patients of age 0-1y, number of entries and amount of test samples exceeding recommended national and EU Phe values. Supplementary Table 1

Supplementary Table 2 Maximal, minimal, and median values of Estonian PKU patients of age 1-2y, number of entries and amount of test samples exceeding recommended national Phe values. Supplementary Table 2

Supplementary Table 3 Maximal, minimal, and median values of Estonian PKU patients of age 2-6y, number of entries and amount of test samples exceeding recommended national Phe values. Supplementary Table 3

Supplementary Table 4 Maximal, minimal, and median values of Estonian PKU patients of age 6-12y, number of entries and amount of test samples exceeding recommended national Phe values. Supplementary Table 4

Supplementary Table 5 Maximal, minimal, and median values of Estonian PKU patients of age 12-18y, number of entries and amount of test samples exceeding recommended national Phe values. Supplementary Table 5

Supplementary Table 6 Maximal, minimal, and median values of Estonian PKU patients of age >18y, number of entries and amount of test samples exceeding recommended national Phe values. Supplementary Table 6
