# Supplementary material for: The evaluation of phenylalanine levels in Estonian phenylketonuria patients during eight years by electronic laboratory records
Source: Mol Genet Metab Rep. 2019 Mar 23;19:100467. doi: 10.1016/j.ymgmr.2019.100467 (PMC6434493; doi:10.1016/j.ymgmr.2019.100467)
Supplement: Supplementary Fig. 1 — Individual yearly average phenylalanine (Phe) values of Estonian phenylketonuria patients. [file mmc1.pdf]

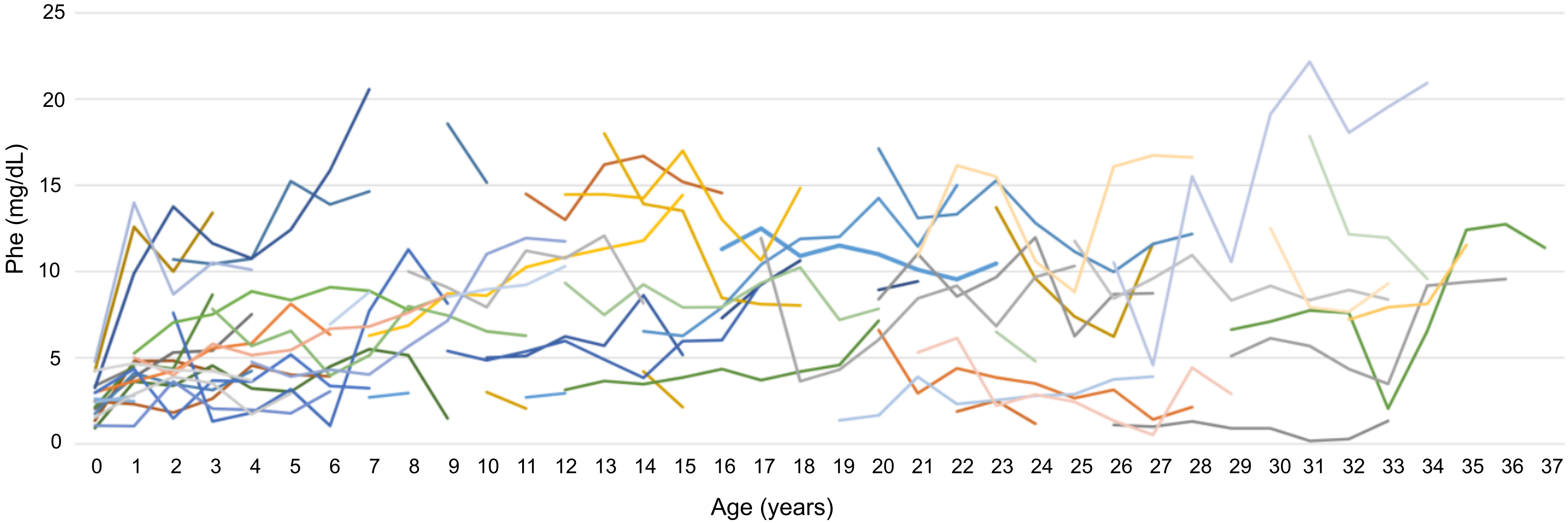

Suppl. Fig. 1. Individual yearly average phenylalanine (Phe) values of Estonian phenylketonuria patients.
