# Supplementary material for: The evaluation of phenylalanine levels in Estonian phenylketonuria patients during eight years by electronic laboratory records
Source: Mol Genet Metab Rep. 2019 Mar 23;19:100467. doi: 10.1016/j.ymgmr.2019.100467 (PMC6434493; doi:10.1016/j.ymgmr.2019.100467)
Supplement: Supplementary Table 1 — Maximal, minimal, and median values of Estonian PKU patients of age 0-1y, number of entries and amount of test samples exceeding recommended national and EU Phe values. [file mmc2.pdf]

Table 1 suppl. Maximal, minimal, and median values of Estonian PKU patients of age 0-1y, number of entries and amount of test samples exceeding recommended national and EU Phe values.

Patient ID	No of entries	min Phe mg/dL	min Phe µmol/L	max Phe mg/dL	max Phe µmol/L	Phe median mg/dL	Phe >/= 6 mg/dL (times)	elevated 6 mg/dL (%)	Phe >/= 4 mg/dL (times)	elevated 4 mg/dL (%)
DI	53	0,9	54	14,3	866	0,9	5	9,4	7	13,2
DC	16	0,9	54	12,6	763	2,7	4	25,0	7	43,8
DJ	35	0,9	54	7,2	436	0,9	1	2,9	1	2,9
DO	50	0,1	6	11,0	664	2,4	5	10,0	16	32,0
CO	7	0,9	54	1,0	61	0,9	0	0,0	0	0,0
CP	35	0,9	54	7,2	436	1,5	2	5,7	8	22,9
DB	34	0,9	54	11,4	690	2,9	4	11,8	12	35,3
DL	14	0,4	23	4,6	276	1,5	0	0,0	4	28,6
DG	48	0,9	54	6,0	363	1,4	1	2,1	3	6,3
DD	44	0,9	54	11,9	720	2,5	4	9,0	13	29,5
DP	23	0,6	39	3,0	179	1,5	0	0,0	0	0,0
DN	53	0,1	8	13,0	786	1,5	8	15,1	10	18,9
DA	42	0,9	54	8,5	515	2,5	4	9,5	9	21,4
DM	45	0,1	7	9,3	564	1,5	5	11,1	10	22,2
DE	51	0,9	54	4,0	242	0,9	0	0,0	0	0,0
DQ	24	0,2	12	9,7	590	0,6	2	8,3	3	12,5
DF	25	0,9	54	5,1	309	0,9	0	0,0	1	4,0
DK	42	0,9	54	11,7	708	4,1	16	38,1	21	50,0
DH	20	0,9	54	11,5	696	2,8	6	30,0	7	35,0
median	35	0,9		9,3		1,5	4	9,0	7	21,4
