# Supplementary material for: The evaluation of phenylalanine levels in Estonian phenylketonuria patients during eight years by electronic laboratory records
Source: Mol Genet Metab Rep. 2019 Mar 23;19:100467. doi: 10.1016/j.ymgmr.2019.100467 (PMC6434493; doi:10.1016/j.ymgmr.2019.100467)
Supplement: Supplementary Table 2 — Maximal, minimal, and median values of Estonian PKU patients of age 1-2y, number of entries and amount of test samples exceeding recommended national Phe values. [file mmc3.pdf]

Table 2 suppl. Maximal, minimal, and median values of Estonian PKU patients of age 1-2y, number of entries and amount of test samples exceeding recommended national Phe values.

Patient ID	No of entries	min Phe mg/dL	min Phe µmol/L	max Phe mg/dL	max Phe µmol/L	Phe median mg/dL	Phe median µmol/L	Phe >/= 6 mg/dL (times)	elevated 6 mg/dL (%)
DI	53	0,2	15	12,9	782	3,1	186	11	20,8
DC	11	8,4	509	20,6	1247	11,2	678	11	100
DJ	32	1,4	85	8,5	517	4,9	299	10	31,3
DO	39	0,5	27	11,5	694	3,3	197	15	38,5
CO	47	0,9	54	12,7	769	2,7	163	9	19,1
CP	38	0,9	54	11,4	690	1,6	97	1	2,6
DB	18	0,9	54	21,2	1283	9,7	587	11	61,1
DL	7	1,6	97	7,9	476	4,7	282	1	14,3
DG	32	1,3	79	9,8	593	3,7	224	7	21,9
DD	37	0,9	54	10,2	618	3,2	194	4	10,8
CN	17	1,7	103	13,1	793	5,0	303	7	41,2
DN	40	0,2	15	10,8	656	1,8	108	4	10
DA	32	1,1	67	12,7	769	4,2	254	8	25
DM	32	0,8	49	8,1	492	1,6	96	5	15,6
DE	54	0,9	54	2,1	127	0,9	54	0	0
CM	19	0,9	54	8,9	539	4,9	297	4	21,1
DF	17	0,9	54	8,4	509	2,5	148	2	11,8
DK	13	9,3	565	19,4	1174	14,2	858	13	100
DH	13	3,1	187	7,9	478	4,7	285	3	23,1
medians	32	0,9	54	10,8	656	3,7	224	7	21,1
