# Supplementary material for: The evaluation of phenylalanine levels in Estonian phenylketonuria patients during eight years by electronic laboratory records
Source: Mol Genet Metab Rep. 2019 Mar 23;19:100467. doi: 10.1016/j.ymgmr.2019.100467 (PMC6434493; doi:10.1016/j.ymgmr.2019.100467)
Supplement: Supplementary Table 3 — Maximal, minimal, and median values of Estonian PKU patients of age 2-6y, number of entries and amount of test samples exceeding recommended national Phe values. [file mmc4.pdf]

Table 3 suppl. Maximal, minimal, and median values of Estonian PKU patients of age 2-6y, number of entries and amount of test samples exceeding recommended national Phe values.

Patient ID	No of entries	min Phe mg/dL	min Phe µmol/L	max Phe mg/dL	max Phe µmol/L	Phe median mg/dL	Phe median µmol/L	Phe >/= 6 mg/dL (times)	elevated 6 mg/dL (%)
DI	105	0,1	7	14,0	848	5,3	322	48	45,7
DC	6	7,4	448	14,2	860	9,9	596	6	100,0
DJ	47	0,3	20	9,4	570	4,4	267	15	31,9
CO	167	0,9	54	15,8	957	2,6	157	33	19,8
CP	31	0,9	54	9,6	581	1,9	115	4	12,9
CK	29	6,4	387	18,8	1138	10,7	648	29	100,0
DB	42	0,9	54	25,1	1520	12,4	753	38	90,5
DL	8	0,5	30	11,1	672	6,9	420	5	62,5
DG	44	7,1	430	7,1	430	3,5	210	5	11,4
CL	7	0,9	54	7,6	460	1,8	109	2	28,6
DD	100	1,8	109	14,8	896	5,1	307	38	38,0
CN	49	1,2	73	13,4	811	7,5	454	40	81,6
DA	31	0,9	54	7,4	446	2,8	170	1	3,2
CJ	45	1,1	67	15,8	957	5,6	339	19	42,2
DE	192	0,1	8	15,1	914	1,8	111	12	6,3
CM	67	0,9	54	10,1	611	5,3	321	21	31,3
CI	15	0,9	54	11,3	684	2,3	139	4	26,7
DF	34	0,9	52	8,5	513	3,3	201	2	5,9
DK	103	4,2	252	15,0	907	9,8	595	97	94,2
DH	18	2,2	134	7,0	426	3,9	239	1	5,9
medians	43	0,9	54	12,4	748	4,7	287	14	31,6
