# Supplementary material for: The evaluation of phenylalanine levels in Estonian phenylketonuria patients during eight years by electronic laboratory records
Source: Mol Genet Metab Rep. 2019 Mar 23;19:100467. doi: 10.1016/j.ymgmr.2019.100467 (PMC6434493; doi:10.1016/j.ymgmr.2019.100467)
Supplement: Supplementary Table 4 — Maximal, minimal, and median values of Estonian PKU patients of age 6-12y, number of entries and amount of test samples exceeding recommended national Phe values. [file mmc5.pdf]

Table 4 suppl. Maximal, minimal, and median values of Estonian PKU patients of age 6-12y, number of entries and amount of test samples exceeding recommended national Phe values.

Patient ID	No of entries	min Phe mg/dL	min Phe µmol/L	max Phe mg/dL	max Phe µmol/L	Phe median mg/dL	Phe median µmol/L	Phe >/= 6 mg/dL (times)	elevated 6 mg/dL (%)
DC	2	10,8	652	23,1	1399	16,9	1025	2	100,0
CO	66	1,3	79	15,0	909	4,2	252	19	28,8
CP	5	1,0	60	7,2	436	2,7	165	2	40,0
CK	20	7,1	430	23,4	1414	15,1	916	20	100,0
DB	9	11,8	716	20,6	1245	15,7	952	9	100,0
CB	3	1,9	115	3,0	182	2,2	133	0	0,0
CA	7	3,4	206	6,6	400	5,1	309	2	28,6
CD	73	0,9	54	11,9	720	5,4	327	30	41,1
CL	7	0,9	54	13,2	799	8,1	493	4	57,1
DD	15	3,2	195	12,6	761	5,8	352	7	46,7
CF	56	1,0	61	20,9	1265	7,9	478	41	73,2
CN	15	5,9	355	11,8	712	8,9	539	14	93,3
DA	14	1,4	86	6,5	394	3,4	206	1	7,1
CE	13	4,5	272	14,3	866	10,1	611	11	84,6
CJ	34	0,9	54	13,3	805	6,3	378	19	55,9
DE	14	1,3	81	10,0	605	2,2	136	1	7,1
CM	32	4,1	246	13,0	790	7,3	441	22	68,8
CI	72	0,9	54	14,5	880	7,1	432	43	59,7
CH	12	4,7	285	12,1	733	8,9	539	9	75,0
medians	14	1,9	115	13,0	790	7,1	432	9	57,1
