# Supplementary material for: The evaluation of phenylalanine levels in Estonian phenylketonuria patients during eight years by electronic laboratory records
Source: Mol Genet Metab Rep. 2019 Mar 23;19:100467. doi: 10.1016/j.ymgmr.2019.100467 (PMC6434493; doi:10.1016/j.ymgmr.2019.100467)
Supplement: Supplementary Table 5 — Maximal, minimal, and median values of Estonian PKU patients of age 12-18y, number of entries and amount of test samples exceeding recommended national Phe values. [file mmc6.pdf]

Table 5 suppl. Maximal, minimal, and median values of Estonian PKU patients of age 12-18y, number of entries and amount of test samples exceeding recommended national Phe values.

Patient ID	No of entries	min Phe mg/dL	min Phe µmol/L	max Phe mg/dL	max Phe µmol/L	Phe median mg/dL	Phe median µmol/L	Phe >/= 10 mg/dL (times)	elevated 10 mg/dL (%)
BH	2	11,3	684	12,5	757	11,9	720	2	100,0
BG	4	7,3	442	11,6	702	8,1	487	1	25,0
BN	8	9,8	593	17,6	1066	16,0	969	7	87,5
CA	15	3,3	200	11,2	678	6,3	381	1	6,7
BL	33	2,2	133	6,6	400	3,5	213	0	0,0
CD	126	0,4	25	15,2	923	5,5	330	8	6,3
BM	23	10,4	630	22,6	1368	14,6	884	23	100,0
BI	143	0,9	54	15,6	944	7,5	454	31	21,7
BJ	16	7,2	436	22,6	1368	13,1	793	13	81,3
CG	1	2,9	178	2,9	178	2,9	178	0	0,0
CF	42	6,0	363	21,5	1302	12,1	730	27	64,3
CE	10	4,3	258	16,9	1023	9,0	545	4	40,0
BF	14	9,0	545	14,8	896	12,0	726	13	92,9
BK	31	4,9	297	11,8	714	8,1	490	9	29,0
CI	7	10,0	607	14,1	854	11,2	675	7	100,0
CH	2	9,9	598	10,7	650	10,3	624	1	50,0
medians	14,5	6,6		14,5		9,7		7	45,0
