# Supplementary material for: The evaluation of phenylalanine levels in Estonian phenylketonuria patients during eight years by electronic laboratory records
Source: Mol Genet Metab Rep. 2019 Mar 23;19:100467. doi: 10.1016/j.ymgmr.2019.100467 (PMC6434493; doi:10.1016/j.ymgmr.2019.100467)
Supplement: Supplementary Table 6 — Maximal, minimal, and median values of Estonian PKU patients of age >18y, number of entries and amount of test samples exceeding recommended national Phe values. [file mmc7.pdf]

Table 6 suppl. Maximal, minimal, and median values of Estonian PKU patients of age >18y, number of entries and amount of test samples exceeding recommended national Phe values.

Patient ID	No of entries	min Phe mg/dL	min Phe µmol/L	max Phe mg/dL	max Phe µmol/L	Phe median mg/dL	Phe median µmol/L	Phe >/= 10 mg/dL (times)	elevated 10 mg/dL (%)
BH	12	6,1	366	13,5	817	10,6	644	10	83,3
BG	22	5,5	333	13,7	831	9,5	575	9	40,9
BC	14	3,7	222	20,4	1235	10,0	602	7	50,0
AF	14	0,2	10	1,8	109	0,9	54	0	0,0
BL	7	3,7	225	7,1	432	4,5	274	0	0,0
AN	41	6,3	382	19,2	1162	12,9	781	35	85,4
BE	12	0,9	54	12,4	751	2,0	119	1	8,3
BB	105	0,3	19	16,2	981	8,5	514	34	32,4
BJ	2	8,0	486	12,5	758	10,3	622	1	50,0
AA	94	0,9	54	19,8	1197	7,5	454	26	27,7
BI	33	7,1	429	18,5	1118	12,8	773	26	78,8
AM	34	0,9	54	10,5	636	2,9	173	1	2,9
AB	41	0,9	54	12,0	726	6,2	375	4	9,8
BM	1	14,8	899	14,8	899	14,8	899	1	100,0
BF	95	0,9	54	14,5	878	6,2	375	14	14,7
BD	2	19,0	1150	19,3	1168	19,1	1159	2	100,0
AD	9	3,4	206	12,5	757	7,2	437	2	22,2
AH	30	5,7	345	15,1	914	8,8	533	9	30,0
BK	5	5,8	349	10,8	654	8,6	522	1	20,0
BA	86	0,9	54	8,1	490	2,5	150	0	0,0
AL	1	14,7	890	14,7	890	14,7	890	1	100,0
JA	24	0,9	54	18,3	1108	15,4	932	19	79,2
AK	6	2,9	176	10,1	611	8,1	490	1	16,7
AI	70	0,2	13	16,0	969	1,6	97	2	2,9
AG	12	5,0	304	18,8	1138	9,3	562	4	33,3
AE	149	0,9	54	26,7	1616	10,6	642	79	53,0
AC	9	9,6	579	23,0	1392	12,5	756	8	88,9
medians	14	3,7		14,7		8,8		4	32,4
